# In Situ EBSD Observation and Numerical Simulation of Microstructure Evolution and Strain Localization of DP780 Dual-Phase Steel

**DOI:** 10.3390/ma18020426

**Published:** 2025-01-17

**Authors:** Yupeng Ren, Shengci Li, Shaohua Feng, Yang Li, Changwang Yuan

**Affiliations:** 1 School of Materials Science and Engineering, Jiangxi University of Science and Technology, Ganzhou 341000, China; 2 Jiangxi Provincial Key Laboratory of High-Performance Steel and Iron Alloy Materials, Ganzhou 341000, China

**Keywords:** dual-phase steel, deformation behavior, martensite, crystal plastic finite element method

## Abstract

To reveal the microstructural evolution and stress–strain distribution of 780 MPa-grade ferrite/martensite dual-phase steel during a uniaxial tensile deformation process, the plastic deformation behavior under uniaxial tension was studied using in situ EBSD and crystal plastic finite element method (CPFEM). The results showed that the geometrically necessary dislocations (GND) in ferrite accumulated continuously, which is conducive to the formation of grain boundaries, but the texture distribution did not change significantly. The average misorientation angle decreased and the proportion of low-angle grain boundaries increased with the increase of strain. At high strain, the plastic deformation mainly occurred in the soft ferrite region within a 45° distribution from the loading direction. In the undeformed state, the texture of the dual-phase steel was characterized by α-fibers and γ-fibers. Interfacial debonding was caused by the accumulation of geometrically necessary dislocations. The fracture morphologies showed that the specimens had typical ductile fracture characteristics.

## 1. Introduction

In recent decades, advanced high-strength steel (AHSS) has been widely used to meet the needs of energy conservation and emission reduction [1]. Dual-phase (DP) steel has been widely used in the automotive industry due to its high strength and good ductility. Typical DP steel is composed of a soft ferrite matrix and hard martensitic islands distributed in the matrix. When subjected to stress, the significant difference between the ferrite and martensite phases results in highly complex microscopic deformation mechanisms. It is found that the initial hardness difference between ferrite and martensite controlled the strain partitioning between the phases, which triggered the microscopic strain localizations at the ferrite–martensite interface and determined the macro-tensile behavior [2].

Hassan et al. [3] experimentally investigated the changes in hardness and strength of the ferrite and martensite regions by using the nanoindentation method. The nanoindentation test showed that the hardness and strength of the ferrite within a single ferrite grain were spatially uneven, and the ferrite near the ferrite–martensite interface was harder than the ferrite inside the grain. Therefore, increasing the hardness of martensite could increase the strength of the material but reduce its ductility [4,5]. The key factors affecting the overall microstructure and mechanical properties of dual-phase steel are the grain characteristics of the two phases. These include the volume fraction of each phase, as well as their morphologies and crystal orientations [6,7,8,9]. Dislocation will interact with each other during movement, such as dislocation delivery, entanglement, etc. These interactions can hinder the movement of dislocations, resulting in the accumulation of dislocations in local areas, creating stress concentrations that promote the formation of shear bands. This strain concentration makes the steel more prone to fracture at the shear zone, reducing the ductility of the steel. The deformation gradient describes the degree of the non-uniformity of deformation within the steel. In the plastic stage, the deformation gradient determines the direction and degree of plastic flow of the steel and affects the yield strength and work hardening behavior of the steel. Texture refers to the preferred distribution of crystal orientation in steel, which results in the obvious anisotropy of mechanical properties. Therefore, although there is a great deal of research on the mechanical properties of DP steel, it is still necessary to understand the microstructural distributions of the strain and stress. The computational method that can accurately reveal the physical mechanism of plastic deformation of DP steel can finally be related to the macroscopic responses of DP steel, which provides a basis for the production and understanding of the microstructures of DP steel.

At present, the in situ electron backscattering diffraction (EBSD) technique is an important means to observe and study the microstructure evolution during deformation [10,11,12]. Compared with interrupted experiments, in situ EBSD has the advantage of high time efficiency, which can capture the microstructural evolution under continuous loading and plastic deformation in real time. Li et al. [13] used in situ EBSD technology to study the plastic deformation behavior of DP600 steel during the uniaxial tensile process. They reported that the plastic strain mainly occurred in the ferritic grains. Tseng et al. [14] studied the relationship between the crack patterns and heterogeneous microstructures of DP780 and DP980 steel by scanning electron microscopy (SEM), semi-in situ deformation experiments, and electron channel contrast imaging (ECCI). They found that deformation dissonance played an important role in restricting the occurrence of microdamage through different strain localization events, and strain localization was determined by the microstructure of the damage site and its environmental microstructure. Wang et al. [15] reported that the crack initiation of 316 L stainless steel during the uniaxial tensile process via in situ EBSD technology was caused by strain concentration and a continuous increase in the dislocation density. In situ EBSD technology can be used to detect and record the crystal structures and morphologies of materials, including dislocation slip, shear bands, and cracks. These microstructural characteristics can be accurately applied in simulation techniques such as the crystal plastic finite element method (CPFEM).

To further deepen the understanding of the effects of the micromechanical plastic deformation and ductile failure behavior of materials, the CPFEM has been widely used [16,17,18,19,20]. In CPFEM, the microstructural characteristics of the material, such as the grain orientation, grain structure (size and morphology), and dislocation slip, can be effectively dealt with [21,22,23]. Kim et al. [24] analyzed the effects of the phase morphology and grain orientation on the interface debonding of DP steels by combining the CPFEM and representative volume element (RVE) method. The simulation results showed that the crystal orientation, martensite morphology, and grain boundary arrangement of ferrite significantly affected the non-uniformity of the stress–strain distribution of DP980 steel under uniaxial tensile conditions. To understand the relative coupling effect and mechanical anisotropy between the hard martensitic region and the soft ferrite matrix, Zhang et al. [25] used a crystal plasticity simulation method for DP steel in different tensile directions. They used different crystal–plastic constitutive parameters for the phases. Knezevic et al. [26] simulated DP steel using a physics-based crystal plasticity model to investigate its behavior under cyclic tensile compression loading conditions. However, there is still much to be studied on how to use CPFEM to investigate the evolution in microstructure and properties during the plastic deformation process of DP steel based on real microstructures.

In this work, the microstructural evolution of DP780 steel during uniaxial tensile deformation was studied via in situ EBSD experiments, including the evolution of the crystal orientation, grain boundaries, texture, lattice rotation, dislocation misorientation, and geometric dislocation density. After the tensile tests, the fracture morphologies were characterized by SEM. Then, the effect of strain localization was studied using CPFEM simulations based on the microstructure. In situ EBSD observation of the microstructural evolution is of great significance for the establishment and verification of CPFEM models. The initial microstructures measured by EBSD were mapped directly to RVEs, and micromechanical simulations were carried out using the CPFEM. The EBSD-mapping RVE model is more realistic because it replicates the actual grain morphology and crystallographic orientation. In addition, since the RVE is modeled at the grain scale, it can be used to accurately study the local stress and strain fields of DP steel after deformation. These methods explore the evolution of the microstructure and the global mechanical responses of local structures. The research results have basic and technical significance for the production and application of DP steel.

## 2. Materials and Methods

### 2.1. Materials

**Figure 1:**
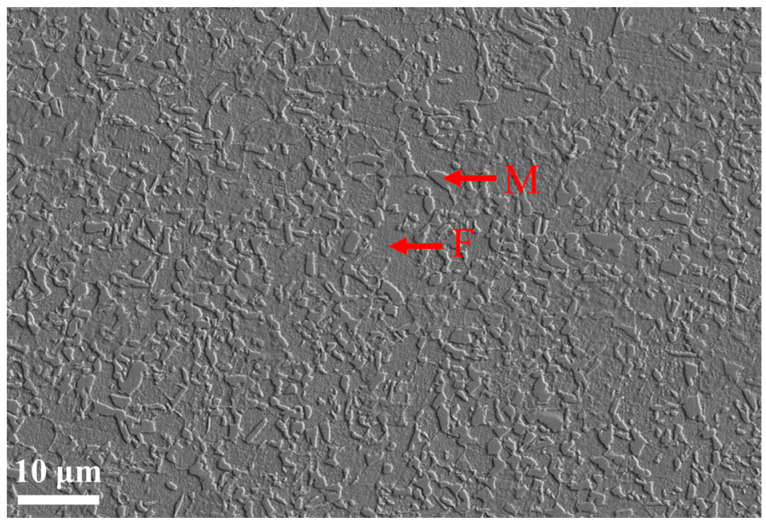
Scanning electron microscopy (SEM) image of DP780. (M: martensite; F: ferrite).

The material used in this paper was a 1.55 mm-thick DP780 steel plate, and the typical chemical composition is shown in Table 1. The SEM image in Figure 1 shows the microstructure of the as-received DP780. The sample surface was prepared by conventional metallographic grinding and diamond polishing, and finally, it was polished with silica gel particles. The surface of the sample was then etched with 2% nitrate alcohol for 10 s. This preparation method yielded a deformation-free surface quality suitable for microstructural characterization. To identify the morphology of the ferrite grains and martensitic islands, the SEM micrograph shown in Figure 1 was used. The martensitic volume fraction of DP780 was 38.4%, the average grain size of ferrite was 5.87 μm, and the average grain size of the martensitic islands was 1.43 μm. The mechanical properties seen from the tensile tests in the rolling direction are shown in Table 2.

### 2.2. In Situ EBSD Measurement

**Figure 2:**
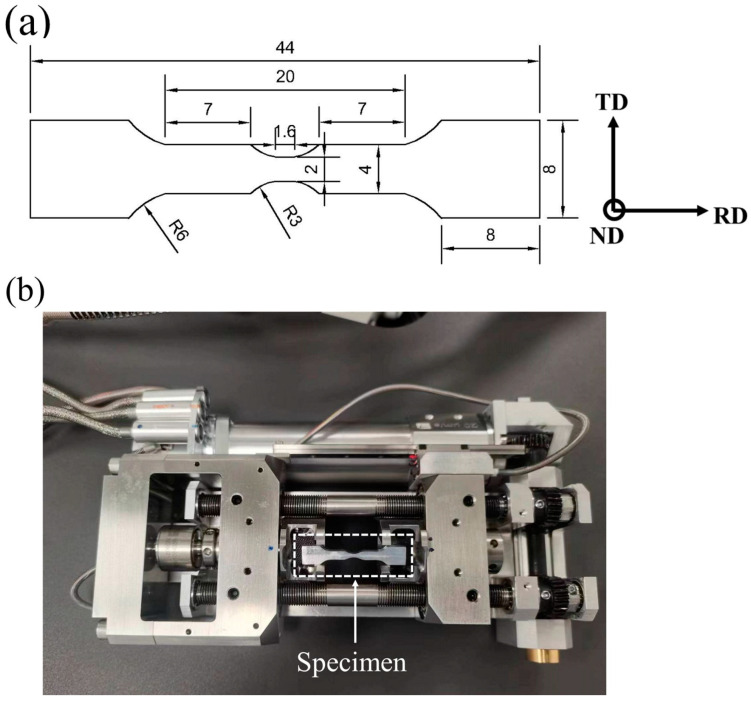
(**a**) Schematic diagram of sample size (unit: mm). (**b**) In situ electron backscatter diffraction (EBSD) system. (RD: rolling direction; TD: transverse direction; ND: normal direction).

A micro-tensile test specimen with a gauge length of 1.6 mm was machined by electrical discharge wire cutting, as shown in Figure 2a. The samples were cut along the rolling direction, and their thicknesses were reduced to 1 mm after surface treatment. The initial microstructures and textures of the samples were characterized by EBSD. Due to the significant strength difference between the ferritic matrix and the martensitic islands, careful sample preparation is required to obtain a high-accuracy EBSD model. The specimen was first mechanically polished with SiC sandpaper, and then it was finely polished with 2.5 and 0.5 μm diamond grinding paste. After polishing, electrolytic polishing was carried out in the mixed electrolyte of 10% perchloric acid + 90% anhydrous ethanol under ultrasonic vibration conditions. The voltage was 20 V, and the polishing time was 30 s. The tensile tests were performed using a 5 kN in situ tensile table, as shown in Figure 2b, mounted on a Thermo Scientific Apreo 2 SEM system and equipped with a high-speed, high-sensitivity, and high-resolution EBSD detector (OXFORD, Tubney Woods, UK). Under a 20 kV acceleration voltage, the normal direction of the sample was scanned with a step size of 0.25 μm, and a region about 200 × 150 μm^2^ was selected as the region of interest. The displacement rate was 1 μm/s, the test was interrupted at different displacements, and the EBSD data of the same region were collected at strain levels of 0, 0.08, 0.16, and 0.24. Secondary electron images at each displacement were also recorded to show the progression of the slip tracer and damage initiation with the strain. The obtained EBSD data were processed using AztecCrystal 2.1 software.

### 2.3. Microstructural Characterization

**Figure 3:**
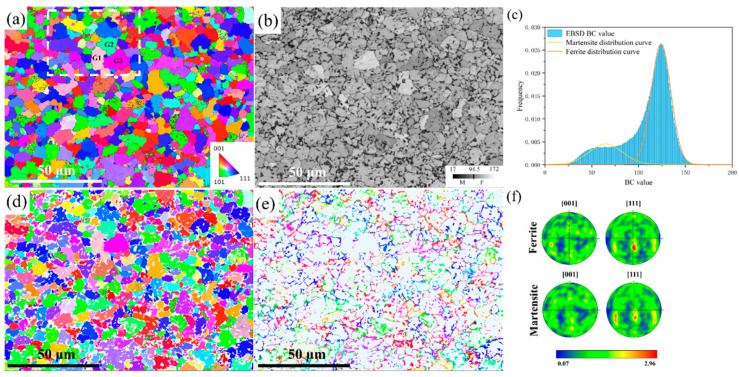
Microstructure of DP780 steel. (**a**) EBSD image of the microstructure on the RD–TD (rolling direction–transverse direction) plane, the white box is the region of interest for simulation. (**b**) Band contrast (BC) map. (**c**) BC distribution and Gaussian fitting of ferrite and martensite phases. (**d**) Separated ferrite phase. (**e**) Separated martensite phases. (**f**) Pole figures of the separated ferrite and martensite phases.

The initial EBSD microstructure of the DP780 steel is shown in Figure 3a, and the ferrite and martensite phases were distinguished based on the inverse pole figure (IPF) and band contrast (BC) map. Martensite and ferrite generally have body-centered cubic (BCC) crystal structures [27,28]. Therefore, it was difficult to distinguish between ferrite and martensite phases based on EBSD data. The phases were classified by the Classify function in AztecCrystal 2.1 software using the BC diagram shown in Figure 3b. Regions with small BC values corresponded to hard martensite particles, and regions with large BC values corresponded to soft ferrite phases. The threshold was determined based on the overlapping region of two Gaussian peaks on the BC frequency distribution curve [13], as shown in Figure 3c. The martensite volume fraction was about 30.5%, and the ferrite volume fraction was about 69.5%. The EBSD IPF maps for the separation of single ferrite and martensite phases are shown in Figure 3d,e. Figure 3f shows the pole figures of the separated ferrite and martensite phases, respectively, and it can be seen that the two phases had similar initial crystallographic textures.

It is worth noting that EBSD images are often subject to inherent limitations, such as signal quality, deterioration of Kikuchi patterns during plastic deformation, difficulty distinguishing between phases with similar crystal structures (e.g., ferrite and martensite), and grain boundary effects or phase–phase interfaces for the precision of measurements. Furthermore, additional uncertainties and inaccuracies can decrease the quality of the images to such an extent that fuzzy logic-based processors must be used. Furthermore, it is worth observing that images similar to each other (in the fuzzy sense) can be merged into a single set of images from which to extract, through fuzzy divergence computations (which turns out to be a measure of distance), a “feature” image by reducing the computational complexity [29]. This possibility should be highlighted, and these interventions could significantly improve the clarity, completeness, and practical applicability of this paper’s results.

### 2.4. Numerical Simulation

**Figure 4:**
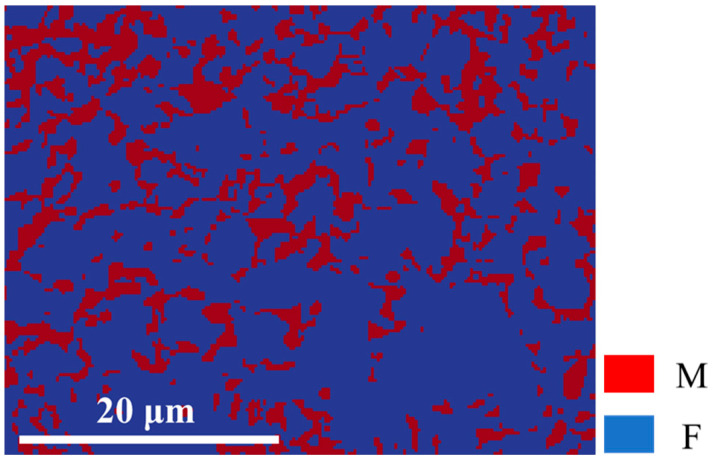
DREAM.3D fitted two-dimensional dual-phase representative volume element (2D-RVE) generated by ferrite and martensitic phases in region of interest.

A two-dimensional (2D) RVE was constructed in the open-source DREAM.3D [30] by taking the regions of interest in the processed EBSD data (the regions selected in the white box in Figure 3a), as shown in Figure 4. The Düsseldorf Advanced Materials Simulation Kit (DAMASK) package [31] was used to simulate the crystal plastic behavior of the DP steels under uniaxial tension. The spectrum solver based on the fast Fourier transform was used to simulate the evolution of the stress and strain states under different strain levels corresponding to the above EBSD measurements. The EBSD data at 0% strain was used as the input microstructure for the simulation. The following approach was followed for the constitutive models as implemented in DAMASK. First, the total deformation gradient F was multiplicatively decomposed to the elastic deformation gradient *F_e_* and the plastic deformation gradient *F_p_*:

*F* = *F_e_ F_p_* (1)

The yielded plastic velocity gradient due to dislocation slip is expressed as:

(2) $$L_{p}=\sum_{\alpha =1}^{N}{\dot{\gamma }}^{\alpha }m^{\alpha }⨂n^{\alpha }$$

Here, ${\dot{\gamma }}^{\alpha }$ is the plastic shear rate, and $m^{\alpha }$ and $n^{\alpha }$ are the slip direction and the normal of the slip system. A phenomenological rate-dependent flow law [32] was adopted to describe the plastic shear rate ${\dot{\gamma }}^{\alpha }$, i.e.,

(3) γ˙α=γ˙0|ταgα|1msign(τα)

with ${\dot{\gamma }}_{0}$ as the reference slip rate, *m* as the rate sensitivity coefficient, and $g^{\alpha }$ as the slip resistance. The evolutions of $g^{\alpha }$ are described as follows [33]:

(4) $$g^{\alpha }=\sum_{\beta }h_{\alpha \beta }{\dot{\gamma }}^{\beta }\mathrm{with}\;h_{\alpha \beta }=h_{0}[q+(1-q)\delta^{\alpha \beta }{\left|1-\frac{g^{\beta }}{g_{\infty }}\right|}^{a}]$$

Here, $h_{0}$ represents the initial hardening modulus of slip systems, $g_{\infty }$ denotes the saturated slip resistance, and *a* is an estimated constant. *q* represents the interaction between slip systems. It is assumed to be 1.0 for coplanar slip systems and 1.4 for non-coplanar slip systems [31]. For linearly elastic metal materials, $T^{e}$ can be computed by Hooke’s law, of which the equation is:

(5) $$T^{e}=\frac{1}{2}C^{e}:(F^{eT}F_{e}-I)$$

Here, $C^{e}$ is the fourth-order elastic tensor. For cubic crystals, $C^{e}$ is formulated with three elastic constants, $C_{11}$, $C_{12}$, and $C_{44}$.

Ferrite and martensite were defined as elastic–viscoplastic deformation phases. The elastic coefficients, shear properties, hardening behaviors, and curve-fitting parameters were derived from the previously published work of Tasan et al. [34], as shown in Table 3.

The uniaxial tensile load in the x-direction was defined using mixed boundary conditions as follows:

(6) $${\dot{F}}_{ij}=\left[\begin{array}{ccc} 1 & 0 & 0\\ 0 & * & 0\\ 0 & 0 & * \end{array}\right]\times 10^{-3}\cdot s^{-1},$$

(7) $${\dot{P}}_{ij}=\left[\begin{array}{ccc} {}* & * & *\\ {}* & 0 & *\\ {}* & * & 0 \end{array}\right]Pa,$$

where ${\dot{F}}_{ij}$ is the macroscopic rate of the deformation gradient, and ${\dot{P}}_{ij}$ is the first Piola–Kirchhoff stress. ${\dot{F}}_{11}=1/s$ for a stretched state and 0 for a restricted state. “*” represents any value during the simulation. All the simulated strain rates were assumed to be 1 × 10^−3^/s. The simulated results were post-processed and then viewed in the open-source Paraview 5.11.1 software.

## 3. Results and Discussions

### 3.1. Microstructural Evolution During Tensile Deformation

**Figure 5:**
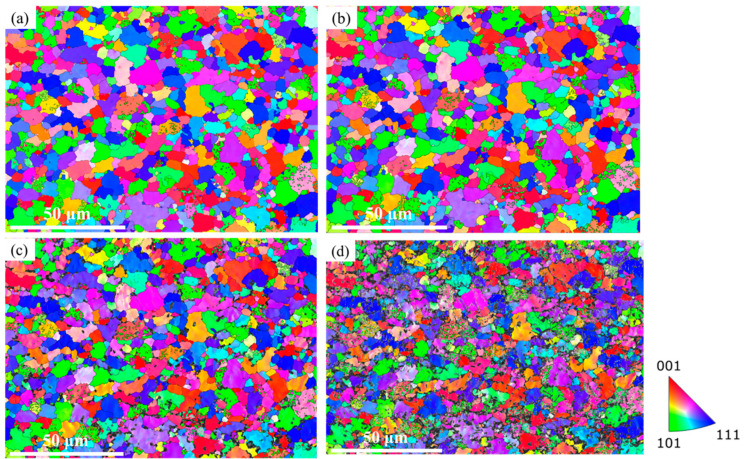
Evolution of grain orientation of DP780 sample under different strain levels: (**a**) 0, (**b**) 0.08, (**c**) 0.16, (**d**) 0.24.

The microstructural evolution of the studied region and some grains during the tensile deformation process was studied by in situ EBSD tensile, as shown in Figure 5. The normal direction of the sample surface was the observation direction, and the horizontal direction was the loading direction. The IPF distributions of the DP780 samples under 0, 0.08, 0.16, and 0.24 strains are shown in Figure 5a–d. It can be seen that the observation area expanded along the loading direction during deformation. When the strain was 0.16, the rotation of the grains could be observed from the IPF map, and the orientation distributions of some grains became uneven and had different degrees of change. At this stage, most of the grains still maintained their original orientations. With the increase in the plastic deformation process, when the strain was 0.24, more grains had uneven orientation distributions. The image quality deteriorated with the increase in the strain, and the black region was zero pixels, which was caused by the increase of the defect and dislocation density caused by the plastic strain. Due to lattice distortion, the quality of the Kikuchi pattern gradually deteriorated, and the size of the black region gradually increased. It can be seen that the grain rotation and grain orientation changed significantly with the increase in the strain, and larger orientation differences occurred for large grains, which evolved into a substructure.

### 3.2. Grain Boundary Evolution

**Figure 6:**
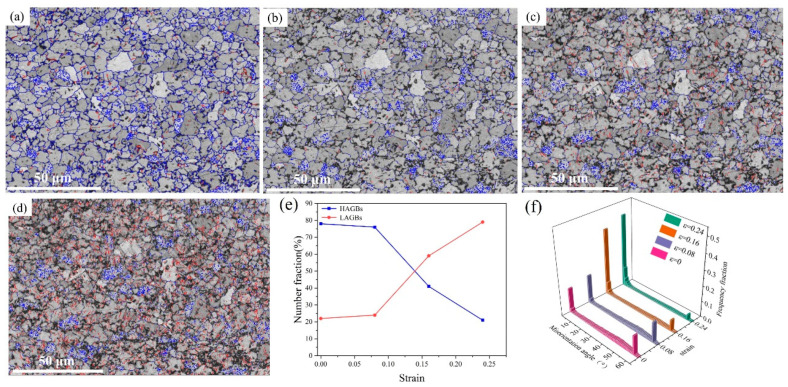
Grain boundary distributions of different strains: (**a**) 0, (**b**) 0.08, (**c**) 0.16, and (**d**) 0.24. (HAGBs and LAGBs are represented by blue and red lines, respectively). (**e**) Fractions of HAGBs and LAGBs. (**f**) Orientation difference angle distributions.

As an interface for separating grains with different orientations, grain boundaries (GBs) have an important effect on the mechanism and mechanical properties of the plastic deformation of metal alloys [35]. The grains with orientation angles of more than 10° are called high-angle grain boundaries (HAGBs), and the grains with 2–10° orientation angles are called low-angle grain boundaries (LAGBs). Figure 6a–d shows the grain boundary distributions at four different deformation levels during in situ tensile tests. HAGBs and LAGBs are represented by blue and red lines, respectively. It can be seen from Figure 6a that most grains exhibited HAGBs (blue line) in the undeformed state, indicating that the microstructure was stable. The new LAGBs produced during the tensile deformation were mainly distributed around the blue HAGBs, and they gradually expanded from the edges of the grains to the interior as the tensile process progressed (Figure 6d). The number of LAGBs increased rapidly at high strain levels, forming major grain boundaries.

Figure 6e shows the evolution of the LAGB and HAGB ratios at different strain levels. It can be seen that in undeformed areas, the proportions of HAGBs and LAGBs were 78% and 22%, respectively. When an external load was applied, the proportion of LAGBs gradually increased, and the proportion of HAGBs gradually decreased. When the strain was 0.24, the proportion of HAGBs decreased to 21% and the proportion of LAGBs increased to 79%. The variation trend of the HAGBs in the deformation process was completely opposite to that of the LAGBs. This may have been because with the increase in the strain, more slip systems within the grains were activated, and the work-hardening phenomenon that occurred during the plastic deformation increased the number of dislocations, which congregated toward the grain boundaries. This promoted the formation of substructures, thereby generating more LAGBs. To minimize the energy of the entire system to maintain stability, dense dislocations were arranged into more ordered states to form new substructures, which formed LAGBs [36].

The waterfall plot in Figure 6f shows the distribution of the grain boundary orientation deviations during tensile deformation. The distribution of orientation deviations before deformation was uneven, with peaks observed around 1.5°, 45.5°, and 59.5°. HAGBs were gradually transformed into LAGBs in the deformation process, resulting in the gradual reduction or even disappearance of HAGB peaks. In this process, a new orientation angle emerged. At the high strain level, the peaks of the orientation difference angles of 45.5° and 59.5° gradually disappeared, and the peak of the dislocation angle of 1.5° gradually increased. This was due to the rotation of grains and the formation of new subgrain boundaries during deformation [37].

### 3.3. Schmid Factor

**Figure 7:**
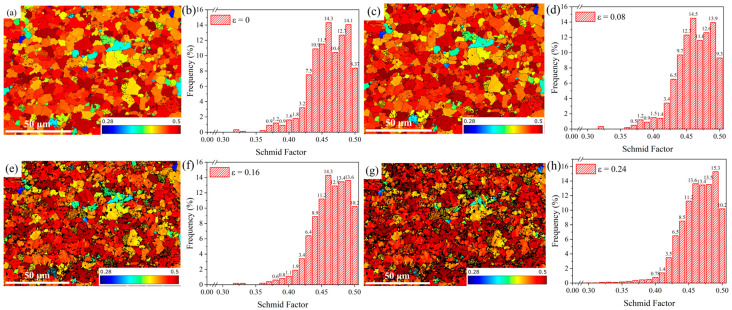
Schmid factor diagram and histograms of Smith factor evolution of the sample under different strains: (**a**,**b**) 0, (**c**,**d**) 0.08, (**e**,**f**) 0.16, and (**g**,**h**) 0.24.

The Schmid factor (SF) plays an important role in material characterization. It reflects the plastic deformation capacity of the material and represents the shear stress applied to a particular sliding system. The higher the SF value is, the higher the decomposition shear stress is, and the corresponding slip system is activated easily under the action of the applied force. DP780 steel is a body-centered cubic (BCC) metal with 12 slip systems. Figure 7 shows the maximum SF values of each slip system in the sample along the RD direction. The SF value was calculated based on the pixels in the EBSD scan area. It can be seen that the particles with high or low SF values in the initial sample were randomly distributed, and the proportion of particles with high SF values was higher. As the strain increased, the proportion of grains with low SF values decreased, as shown in Figure 7c,e,g. Moreover, the grain orientation changed with low SF values.

To further accurately analyze the SF, the distributions of the SF values were statistically analyzed. The distributions of SF values under different strains are shown in Figure 7b,d,f,h. The peak value of the SF in the deformation process was between 0.45 and 0.5, showing a gradually increasing trend. The average SF value without deformation was 0.4. When the strain increased to 0.08, 0.16, and 0.24, the SF values were 0.4, 0.41, and 0.42, respectively. Correspondingly, the proportion of SF values in the 0.45–0.5 range increased from the initial 71.34% to 77.27%. This showed that the SF factor increased with the increase in strain.

When the sample was loaded in a certain direction, only the particles with the highest SF value were activated and deformed first. During the deformation process, the sliding plane and direction of the activated sliding system rotated and converged to orient in the direction of shear. When the strain was less than 0.08, elastic deformation occurred in the sample, so the change of the SF value was not evident. When the strain was more than 0.16, grain deformation occurred, and multiple slip systems formed simultaneously or alternately.

It can be seen that individual grains were deformed by the activation and rotation of the slip system to release stress and maintain the continuity of the specimen. To activate the slip system, the hard-oriented grains continued to rotate during deformation to obtain a favorable orientation, while the adjacent soft-oriented grains underwent coordinated rotation to ensure the continuity of all the grains.

### 3.4. Texture Evolution

**Figure 8:**
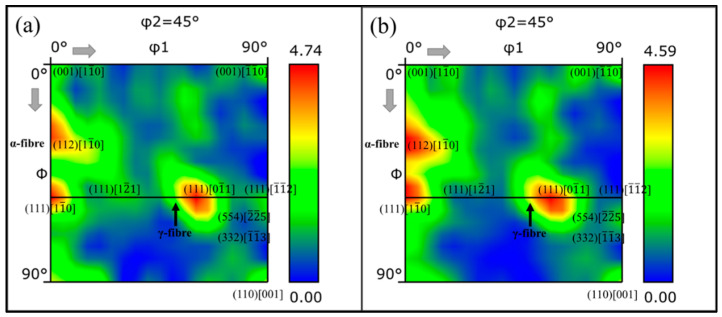
Orientation distribution of DP780 steel under strains of (**a**) 0 and (**b**) 0.24.

In polycrystalline materials, the grain orientation is mainly concentrated near a specific orientation position, which is called the preferred orientation. This phenomenon is called the texture in crystallography. The macroscopic properties of polycrystalline materials were affected by two factors: the single-crystal anisotropy and the grain orientation distribution. Figure 8 shows the orientation distribution function (ODF) for the φ2 = 45° section, which was used to determine whether the texture changed after tensile deformation, indicating grain rotation. The texture of the DP steels could be characterized by the α fiber, which comprises orientations with RD//<110>, and the γ fiber with <111> orientations parallel to the sheet normal direction ND. Texture orientations of (112)[1$\bar{1}$0] and (111)[1$\bar{1}$0] were the main components of the α-fibers, while the orientation of (111)[1$\bar{1}$0] was the main component of the γ-fibers. During the tensile process, the textures of the α-fibers and γ-fibers decreased overall and became denser, and the grain orientations were closer to (112)[1$\bar{1}$0] and (111)[1$\bar{1}$0].

### 3.5. Grain Rotation

**Figure 9:**
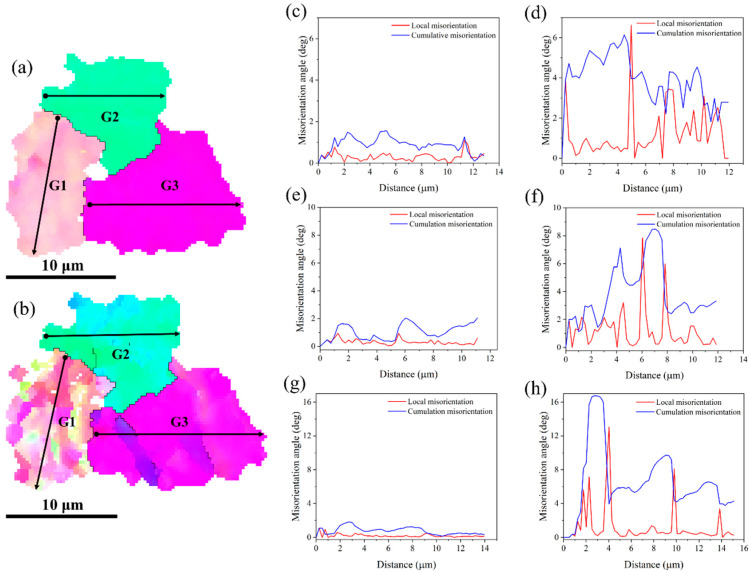
Misorientation changes of each grain during in situ tensile tests. (**a**) Unstrained; (**b**) 0.24 strain. Distribution of misorientation deviation of different grains under (**c**,**e**,**g**) unstrained and (**d**,**f**,**h**) 0.24 strain: (**c**,**d**) G1,(**e**,**f**) G2,(**g**,**h**) G3.

To study the rotation of the grains during deformation, three typical grains (G1, G2, and G3) were selected from the initial IPF diagram, as shown in Figure 9. By tracing the IPF map of these grains during the in situ tensile process, it can be seen that the orientations of the grains changed with the increase in strain. Along the loading direction, the grain sizes of G2 and G3 increased from 9.89 and 12.07 μm to 10.08 and 12.31 μm, respectively, while that of G1 increased from 6.71 to 7.45 μm.

The influence of strain on the misorientation angles was further analyzed, and the point-to-point (cumulative) misorientation and point-to-point (local) misorientation profiles were calculated along the lines in Figure 9. Figure 9c,e,g shows the misorientations of G1, G2, and G3 in an undeformed state, respectively. The cumulative and local orientation differences within the grains of G1, G2, and G3 did not exceed 3°. Figure 9d,f,h shows the orientation deviations of G1, G2, and G3 at a strain of 0.24, respectively. The local/cumulative orientation deviation within the grains was increased, and the maximum cumulative orientation deviation in G3 was nearly 17°, indicating that the progressive rotation of the subgrains was sensitive to strain, and the orientation difference continued to accumulate until regular grain characteristics appeared. This indicated that the evolution of the grain orientation during deformation was closely related to the initial orientation. Grain rotation may be the result of a large strain due to stress triaxiality and the plane stress state due to the sample size. With the increase in strain, the grain orientation needed to be adjusted to activate the slip system to ensure the coordinated deformation between adjacent grains.

### 3.6. Geometrically Necessary Dislocation (GND)

**Figure 10:**
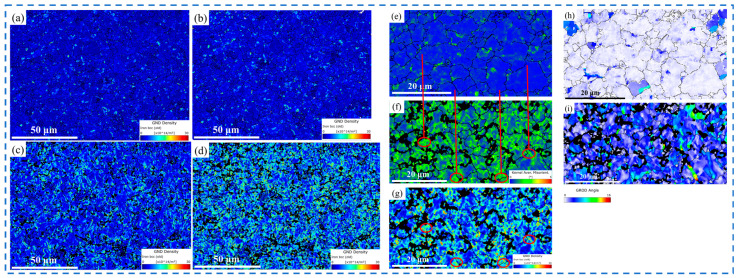
Changes in density of geometrically necessary dislocation (GND) of specimens under strains of (**a**) 0, (**b**) 0.08, (**c**) 0.16, and (**d**) 0.24. (**e**) Non-strain KAM diagram; (**f**) KAM diagram for sample under 0.24 strain and (**g**) GND diagram. (**h**) Grain reference orientation deviation (GROD) for sample under no strain and (**i**) GROD for sample under 0.24 strain.

The plastic deformation process involves dislocation that leads to the formation of geometrically necessary dislocations (GNDs). Strong strain localization is usually accompanied by a high GND density. The GND density can be calculated from the orientation gradient of the crystals. The minimum GND density can be obtained from 2D EBSD data. AztecCrystal 2.1 software was used to calculate the minimum GND density from the EBSD data. In our analysis, the applied Kernel size was a 3 × 3 square, the maximum orientation deviation was 5°, and the Burgers vector was 1/2<111>. Figure 10a–d shows the GND density plot derived under a gradually increasing tensile plastic strain. Approximately 1100 grains were included in each plot to gather a statistically representative dataset. Martensite particles (the low image quality (dark) regions in Figure 3b) were not taken into account when calculating the GND density. The GND density plot shown in Figure 10a–d was only related to the variation of the GND in the ferrite grains. Increasing the plastic strain caused the average GND density to increase from 2.36 × 10^14^ m^−2^ to 8.65 × 10^14^ m^−2^. In addition, since the diffraction pattern quality decreased with the plastic deformation, this resulted in fewer points being available for GND calculations.

With the increase in plastic strain, the GND density near the ferritic grain boundaries and the ferritic martensite grain boundaries increased. For deformed materials, due to the dislocations sliding inside the grains, the dislocation accumulation and accumulation near the grain boundaries resulted in a high GND density near the grain boundaries. These were regions of low strain compatibility between the deformed grains, where lattice rotation occurred at a small distance from the boundary to accommodate the necessary strain in each grain. Figure 10e–g shows the kernel average misorientation (KAM) diagram of the DP steel before deformation and the KAM and GND density diagrams of the DP steel after 0.24 deformation, respectively. The orientation deviation gradient increased with the increase in deformation. Regions with higher KAM values coincided with regions with higher GND densities. This is consistent with several previous studies [38,39,40]. In DP780, the ferrite grains were larger than the martensitic islands, and the ferrite volume fraction was larger. Such a microstructure means that ferrite had great plasticity. Because the yield strength of ferrite is much lower than that of martensite, plastic strain occurred at the interface during plastic deformation, and GND compensation strain was incompatible in ferrite near the interface.

Grain reference orientation deviation (GROD) diagrams can also be used to characterize the uneven distribution of deformation in the microstructure. These data have similar information to those showing the GND content but clearly show differences in the grain-to-grain behavior, so it is worth analyzing GROD information in addition to GND information. Figure 10h,i shows DP steel before deformation and after 0.24 deformation. The maximum value of the GROD is related to the development degree of deformation inhomogeneity [41]. It can be seen that the strength of the GROD in the deformed steel was significantly enhanced, and its distribution was uneven. The GROD distribution was generally uniform in the undeformed steel. When the plastic strain was 0.24, the higher GROD regions were close to the grain boundaries. The smaller the particles were, the higher the GROD values were. By comparing with Figure 10f, it can be seen that higher GROD regions were associated with a higher GND. The compatibility requirements changed during plastic deformation, resulting in a change in the GROD distribution. Due to the presence of martensite in the ferrite matrix, the microscale deformation gradient between the ferrite and martensite was larger.

### 3.7. Simulated Stress–Strain Distribution

**Figure 11:**
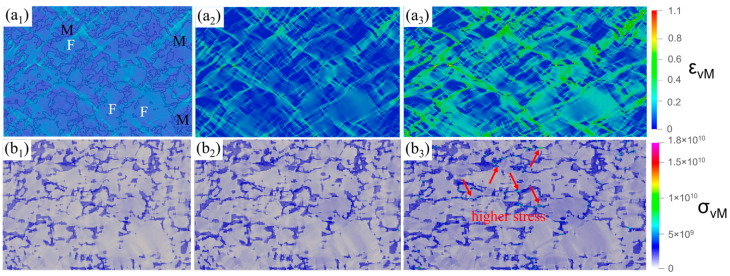
Strain distribution when ε = (**a_1_**) 0.05, (**a_2_**) 0.10, and (**a_3_**) 0.20. Stress distribution when ε = (**b_1_**) 0.05, (**b_2_**) 0.10, and (**b_3_**) 0.20. (The red arrows in the figure indicate higher local stresses).

**Figure 12:**
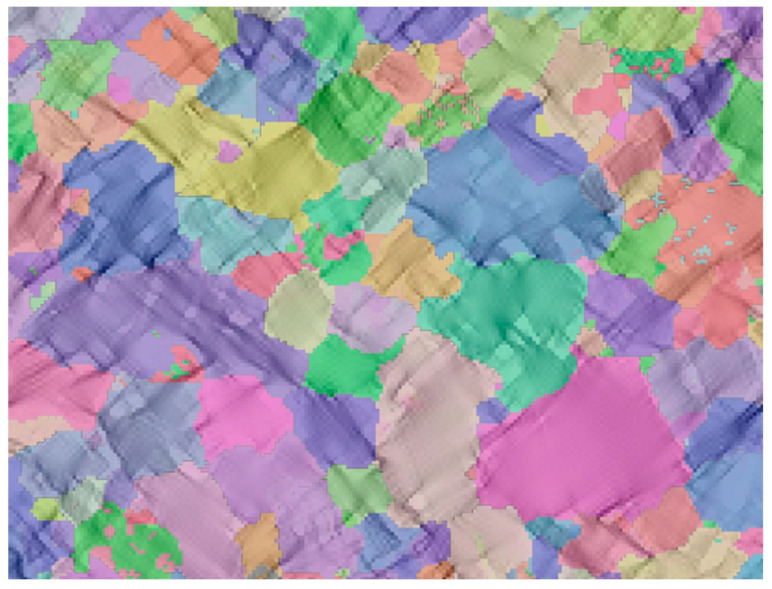
Overlap between EBSD map and the results of numerical simulations under the applied strain conditions of 0.2.

Figure 11 depicts the simulated stress–strain development of the microstructure under the applied strain conditions of 0.05, 0.1, and 0.2. Most of the applied stress was borne by the martensitic phase instead of the ferrite phase. Moreover, in the narrow martensite region parallel to the loading direction, the local stress was higher, as shown by the arrow in Figure 11(b3). Under the small macroscopic applied strain before necking, the maximum plastic strain occurred between the tightly distributed martensitic particles located between the larger ferrite grains. Therefore, these regions were more likely to trigger local deformation belts. It can be seen from Figure 11(a2,a3) that with further application of macroscopic strain, the initial shear bands spread in these regions. These shear bands were generally considered to be initial shear bands, but not all initial shear bands caused the material to fail at high strain values. In other words, only some initial shear bands caused the material to break down. Shear bands that faced fewer obstacles along the growth path could spread freely along a path consistent with the direction of the maximum shear stress. The shear band in the specimen at a 45° angle to the tensile direction was the main shear band. If the path of the shear band propagation was blocked, the shear band was suppressed. It can also be seen from Figure 11(a3) and Figure 12 that the region with a high degree of strain localization in the principal shear zone was located at the junction of ferrite and martensite. High-deformation gradients existed in these regions, leading to the formation of high-strain localization. This observation was in good agreement with Figure 10f, where areas inside the ferritic grains hardened during tensile deformation, while areas near the ferritic–martensite interface softened during deformation.

It can be concluded that the stress–strain distribution during the plastic deformation process of DP steel can be simulated by the in situ EBSD and crystal plastic finite element method, and the effects of the microstructure characteristics of DP steel on the stress–strain distribution can be further investigated. The microstructural strain and stress partitioning could govern the overall mechanical behavior of DP. Therefore, it is a promising method to optimize the microstructure of DP steel by regulating the rolling process and heat treatment process during the production process to obtain the required properties.

### 3.8. Fracture Profile and Morphology

**Figure 13:**
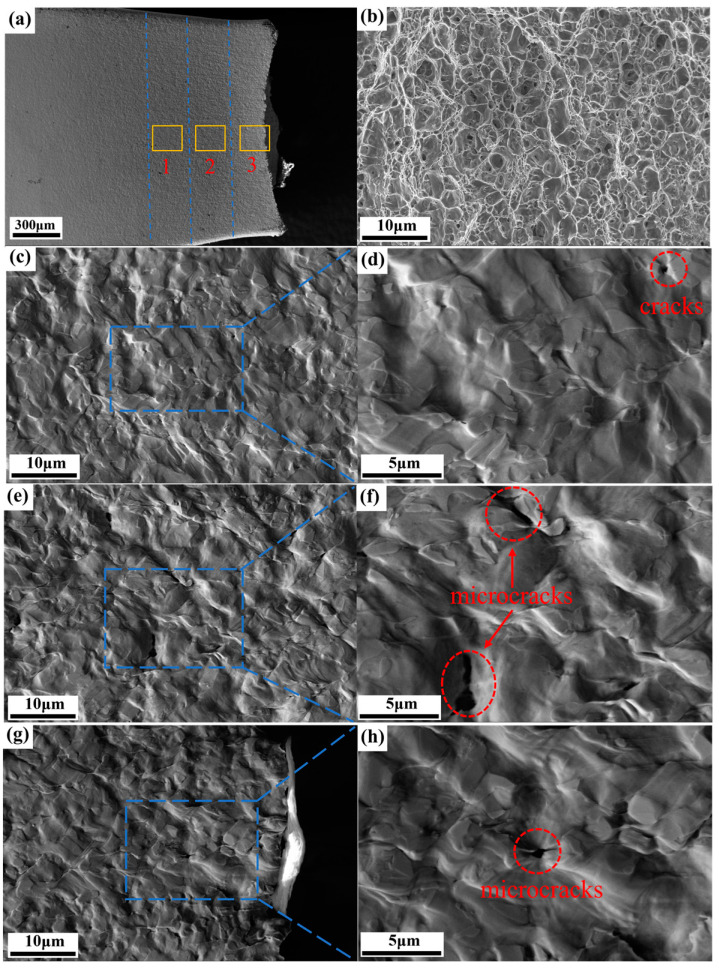
(**a**) SEM images of tensile fracture profile (Boxes 1, 2, and 3 indicate the position of the fracture from far to near); (**b**) fracture morphology, (**c**) region 1 in (**a**); (**d**) enlargement of the box in (**c**); (**e**) region 2 in (**a**); (**f**) enlargement of the box in (**e**); (**g**) region 3 in (**a**); (**h**) enlargement of the box in (**g**).

Figure 13 shows the post-fracture microstructures at different necking positions observed from the center of the fracture along the tensile direction. Figure 13a shows the fracture profile and three regions observed along the section direction. As shown in Figure 13b, the fracture morphology was composed of many dimples of varying sizes, with an approximate equiaxial shape. The micropore nucleation during the tensile fracture of the DP steel was related to the ferrite–martensite fracture itself, and a typical ductile fracture was observed on the fracture surface. These results suggested that as the pores grew, ligaments between the pores were located, and subsequently, the pores merged. This eventually led to a fracture dominated by debonding voids at the ferrite–martensite interface. Figure 13c–h shows the SEM images of three regions under different degrees of necking. As shown in Figure 13d, holes (marked with red circles) caused by ferrite–martensite interfacial debonding were observed in areas far from the fracture. In Figure 13f,h, microcracks were observed in the ferritic matrix, indicating the occurrence of cavity coalescence. These results showed that at the near end of the fracture, the martensite involved in the late coordinated deformation was significantly elongated. Part of the martensite broke, the cracks expanded to the interface between the martensite and ferrite, the martensite gradually peeled off, and, finally, voids formed. The martensite in dual-phase steel is lath martensite with low carbon content, which can be self-tempered, and the carbide is evenly distributed. The dislocation distribution in the cellular dislocation substructure is uneven, and there is a low-density dislocation zone, which provides room for dislocation movement. The dislocation movement can ease the local stress concentration, so the martensite has a certain plasticity. With the increase of stress, it is easy to lead to the sliding and cracking of laths in martensite, resulting in the breaking of some martensite regions. Due to the significant differences in properties between martensite and ferrite, incompatible strains between the two phases will lead to stress concentration at the F/M phase interface, resulting in the formation of cracks. At the far end of the fracture, the number of voids decreased, and the degrees of martensite deformation and fracture decreased [42].

## 4. Conclusions

In this paper, the deformation behaviors of DP780 steel were investigated by in situ EBSD tensile tests and numeral simulations. The main conclusions are as follows:

(1) The deformation of grains in DP steel was not uniform and was related to the Schmid factor value of individual grains. The content of LAGBs in the steel increased with the increase in the tensile deformation.
(2) The grain rotation of DP780 steel during deformation was closely related to its initial orientation. Grain rotation may be the result of the large strain caused by stress triaxiality and the plane stress state caused by the sample size, which was conducive to improving the resistance and plastic deformation ability of DP steel.
(3) The average GND density increased with the increase in plastic deformation. The distributions of the GNDs in the ferrite grains varied, and a higher GND content was observed at the grain boundaries.

## Figures and Tables

**Table 1:** Chemical composition of DP780 steel (wt.%).

C	Mn	Si	P	S	Nb	Ti	Cr	Fe
0.13	1.85	0.36	0.023	0.0043	0.015	<0.005	0.38	Bal.

**Table 2:** Tensile properties of DP780.

Yield Strength/MPa	Ultimate Tensile Strength/MPa	Elongation/%	Yield Ratio
477	825	27.0	0.5782

**Table 3:** Crystal–plastic constitutive parameters of ferrite and martensitic phases.

Property	Value (Ferrite)	Value (Martensite)	Unit
C_11_	233.3 × 10^9^	417.4 × 10^9^	Pa
C_12_	235.5 × 10^9^	242.4 × 10^9^	Pa
C_44_	128.0 × 10^9^	211.1 × 10^9^	Pa
$\dot{\gamma_{0}}$	1 × 10^−3^	1 × 10^−3^	ms^−1^
$S_{0\cdot [111]}$	95 × 10^6^	406 × 10^6^	Pa
$S_{\infty \cdot [111]}$	222 × 10^6^	873 × 10^6^	Pa
$S_{0\cdot [112]}$	96 × 10^6^	457 × 10^6^	Pa
$S_{\infty \cdot [112]}$	412 × 10^6^	971 × 10^6^	Pa
h_0_	1 × 10^9^	563 × 10^9^	Pa
$h_{\alpha \beta }$	1.0	1.0	
n	20	20	
ω	2.25	2.25	

Reproduced with permission from Elsevier.

## Data Availability

The original contributions presented in the study are included in the article, further inquiries can be directed to the corresponding author.
